# Calculation of blast hole charge amount based on three-dimensional solid model of blasting rock mass

**DOI:** 10.1038/s41598-021-04615-8

**Published:** 2022-01-11

**Authors:** YingXian Chen, PengFei Wang, Jian Chen, Meng Zhou, HongXia Yang, JiaYing Li

**Affiliations:** College of Mining, Liaoning Technical University, Fuxin, 123000 China

**Keywords:** Coal, Computational science

## Abstract

The development and use of intelligent drilling rigs make it available to obtain accurate lithology data of blast drilling. In order to make full use of drilling data to improve blasting efficiency, the following research was carried out. First, a database is established to manage and store the blast hole data recognized by the intelligent drill. Secondly, the blast hole lithology data is taken as a sample, and the inverse distance square method is used to interpolate the blasting range's solid elements to generate a three-dimensional solid model of the blasting rock mass. Afterward, the blasting range polygon and stope triangle grid are used successively in the solid model to obtain the cut 3D solid model of the blasting rock mass; finally, the blast hole charge is calculated based on the cut 3D solid model of the blasting rock. The C++ programming language is used to realize all the blast hole charge amount processes based on the three-dimensional solid model of the blasting rock mass. With the application example of No. 918 bench blasting of Shengli Open-pit Coal Mine in Xilinhot, Inner Mongolia, the blast hole charge amount in the blasting area is calculated and compared with the results of single hole rock property calculation, the results show that the blast hole charge calculated by three-dimensional rock mass model can be effectively reduced.

## Introduction

An intelligent drilling rig can accurately obtain the lithology data of the blasting area. The urgent problem that needs to be researched, developed, and solved is how to make full use of the lithology data of the blasting area, accurately calculate the volume of various rock masses, and innovate the calculation method of the blast hole charge to improve the blasting effect and reduce the blasting cost. Currently, many scholars have done much research on intelligent lithology recognition. Guohe Li et al. proposed a new lithology identification method, which uses multiple sampling points of geological data as input. From the comparison of the experiment, it can be seen that the SGAN-G algorithm can effectively use unlabeled data to achieve higher lithology prediction accuracy^1^. Sahoo et al. established and proposed the traditional artificial neural network (ANN-GDM) based on gradient descent momentum training and the traditional genetic algorithm (GA) based on the genetic algorithm ANN artificial neural network (ANN-GA) method^2^. Santos et al. evaluated the advantages of methods based on Neural Network Classifier Ensembles—sets of neural networks working in a cooperative way to achieve a consensus decision- in the solution of the lithology recognition problem^3^. Shao et al. proposed that the BP neural network algorithm has the characteristics of high identification accuracy and fast identification speed, which is suitable for identifying geological research such as petrology, deposit prediction, rock and mineral identification, etc.^4^. Zhang et al. proposed a lithology identification method for the volcanic reservoir, which deals with the log data by combining the principal components analysis and the SOM neural network method to effectively improve neural network performance^5^.

Many scholars have conducted much research on 3D solid modeling. Sun presents a fast self-adaptive and improved inverse distance-weighted interpolation method depending on the borehole sampling data features^6^. Song et al. created a semi-automatic 3D modeling and visualizing method for complex geological bodies by combining typical GIS systems with 3D modeling software such as ArcGIS and SketchUp^7^. Liu introduced the concept of “correlation distance” to analyze the correlations between geological borehole elevation values and calculated the correlation distances for each stratum elevation^8^. Jia et al. proposed an improved anisotropy-based, multiscale interpolation method applied to effectively model coal seam surface^9^. Che proposed a Three-Dimensional geological modeling of coal seams using the weighted Kriging method and multi-source data^10^. In order to bridge the currently existing gap between AutoCAD solid models and the grid modeling realm, Marschallinger established the Visual LISP program that converts AutoCAD solid models into voxel arrays^11^. However, due to the immature development of intelligent drilling rigs, no expert has been engaged in 3D solid modeling of blasting rock mass.

In order to reduce the cost of blasting while improving the blasting effect, many scholars and engineers have done plenty of research on the auxiliary calculation method of blast hole charge. Li accorded to the equivalent resistance theory of rock breaking for the columnar charge, the Livingston theory of blasting crater and the line-loaded density was applied to deduce the theoretic calculation formula of blast hole depth in the progressive spiral cut^12^. Wang took Paishanlou gold mine construction in Liaoning as an example, carried out analysis and calculation of determining methods and practice data of various deep-hole blasting parameters combined with general engineering conditions such as resistance line of the undercarriage, pitch, space between rows, ultradeep value of drilling, and charge quantity of blast hole^13^. Adhikari et al. summarized the experience and methods for calculating the specific charge for open-pit blast design and found that the desired degree of fragmentation, distribution, transfer, and utilisation of explosive energy greatly influences the specific charge^14^. Shim estimated the rock factor from geologic data, and a sequential indicator simulation technique predicted its 3-D spatial distribution. The entire quarry in question was classified into three types of rock mass, and an optimum blasting pattern was proposed for each type based on the 3-D spatial distribution of the rock factor. It can be concluded that it is possible to design a blasting pattern to achieve a minimum production cost in large-scale quarrying operations by predicting rock fragmentation based on the 3-D spatial distribution of the rock factor^15^. At present, most of the commonly used methods for calculating blast hole charge are based on the lithology data or experience of exploration boreholes to obtain lithology distribution. Due to the high distribution density of exploration boreholes, the lithology distribution in the blasting area is inaccurate, which often causes excellent deviations to the blasting design. The way to calculate the blast hole charge based on the 3D solid model of the blasting rock mass is to make full use of the lithology data of the blasted rock mass to establish an accurate 3D solid model of the blasted rock mass and calculate the blast hole charge based on the accurate 3D solid model, improving the blasting effect and reducing the blasting cost.

This paper consists of four parts: the first part mainly introduces lithology identification and the establishment of blast hole database, the second part mainly introduces the calculation method of blast hole charge amount, and the third part mainly presents the calculation method based on the three-dimensional solid model of blasting rock mass. Finally, the calculation method of charge quantity is applied in the Xilinhot Shengli Open-pit Coal Mine in Inner Mongolia Autonomous Region, China, as an example.

## Lithology identification and establishment of blast hole database

The mine rock blast hole data collected from the intelligent drilling rig is stored in files, and a database is established for storage and further management and application of blast hole data.

### Blast hole data structure

The first seven lines of the data collected from the smart rig record the drilling number, rig status, rig number, boot time, longitude, latitude and elevation, respectively. Lines 8 to 16 are records of a section of rock column. These data include drilling number, drilling depth, rotation speed, rotation pressure difference, pressurization pressure 1, pressurization pressure 2, drilling speed, wind pressure, and identified lithology. Subsequently, this record data will be circulated for each rock pillar until the blast hole is completed. The data listed in Table 1 is the data of a section of rock pillar with blast hole number 0620171118170059 collected by the intelligent drill. These data constitute the blast hole data files, but these data files are not convenient for management and further application. It is necessary to establish a database to store and manage this data.

**Table 1:** Blast hole data.

Serial number	Field name	Data
1	Drilling number	0620171108111035
2	Rig status	0
3	Rig number	6
4	Boot time	20171108111035
5	Longitude	116°0′6.0″
6	Latitude	43°59′57.0″
7	Elevation	915.76 m
8	Drilling number	0620171108111035
9	Drilling depth	425 cm
10	Rotation speed	120 r/min
11	Rotation pressure difference	50 bar
12	Pressurization pressure 1	54 bar
13	Pressurization pressure 2	7 bar
14	Drilling speed	4 cm/s
15	Wind pressure	4 bar
16	Identified lithology	1

### Design of blast hole database

**Figure 1:**
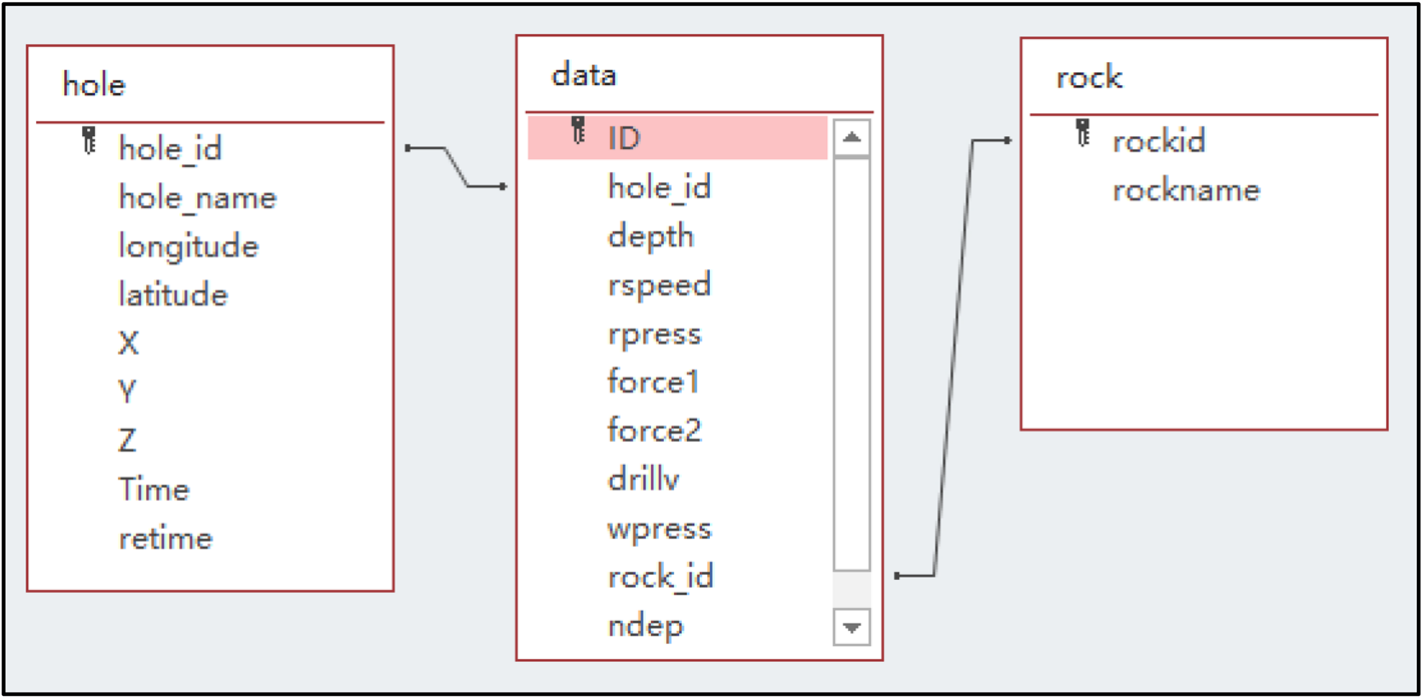
Relationship between the data tables.

Use Microsoft's Access to build a blast hole database and establish three data tables in the database: blast hole table (hole), blast hole data table (data), and lithology table (rock) used to store data collected from intelligent drilling rigs. The relationship between these three data tables is shown in Fig. 1. The blast-hole table (hole) is associated with the blast-hole data table (data) via the hole_id, and the lithology table (rock) is associated with the blast-hole data table (data) via the rock_id.

### Blast hole data extraction

When extracting the blast hole data from the data files into the blast hole database, it is necessary to convert the blast hole coordinates expressed by the longitude and latitude of the blast hole into x, y coordinates. The obtained blast hole table, blast hole data table and lithology table are shown in Tables 2, 3 and 4, respectively.

**Table 2:** Blast hole table.

Hole_id	Longitude	Latitude	X	Y	Z	Retime
ZK2023	116°0′15.0″	43°59′48.0″	19,477.48	74,464.33	916.61	2018/9/13 10:10
ZK2024	116°0′17.0″	43°59′48.0″	19,480.17	74,458.97	916.19	2018/9/13 10:10
ZK2025	116°0′14.0″	43°59′48.0″	19,482.85	74,453.60	916.03	2018/9/13 10:10
ZK2026	116°0′12.0″	43°59′49.0″	19,485.54	74,448.24	915.97	2018/9/13 10:10
…	…	…	…	…	…	…
ZK2027	116°0′15.0″	43°59′49.0″	19,488.22	74,442.87	916.08	2018/9/13 10:10

**Table 3:** Blast hole data sheet.

ID	Hole_id	Depth (m)	Rspeed (r/min)	Rpress (bar)	Force1 (bar)	Force2 (bar)	Drillv (cm/s)	Wpress (bar)	Rock_id	ndep
25971	ZK2023	16.62	90	55	43	7	6	5	3	21
25972	ZK2023	4.68	75	46	30	7	3	4	3	1
25973	ZK2023	5.04	105	55	40	8	7	4	3	2
25974	ZK2023	5.37	120	39	30	7	3	4	3	3
…	…	…	…	…	…	…	…	…	…	…
25975	ZK2023	5.74	90	49	40	7	6	5	5	4

**Table 4:** Lithology data.

Rock-id	Rock name
1	Mudstone
2	Siltstone
3	Sandy mudstone
4	Grit stone
5	Carbon mudstone
6	coal
7	Calcareous or siliceous cemented conglomerate

## Calculation method of blast hole charge amount

Nowadays, rock blasting has been widely used in civil, water conservancy, hydropower, transportation, and other construction engineering fields^16–18^. In open-pit mining, blasting operation is one of the essential business processes of open-pit mining technology^19–23^, which is related to the production capacity and economic benefits of open-pit mining. Therefore, in the production blasting design, it is necessary to be as accurate and efficient as possible to achieve a better blasting effect to meet the requirements of high-efficiency production on site. Therefore, the calculation of the blast hole charge amount is fundamental, and the conventional formula for the calculation of the blasting design is as follows^24^:

1 $$Q = qawh,$$

where *Q* is the blast hole charge amount, kg; *q* is the powder factor, kg/m^3^; *a* is the spacing, m; *w* is the burden, m; *h* is the bench height, m.

From the above calculation formula of blasting charge amount, to make the calculated total blasting charge amount close to the actual charge amount required for high-efficiency blasting, the selection of unit explosive consumption *q* is required to be very strict. This is very difficult for companies that do not have long-term production experience data as parameters. The improper value of the unit consumption of explosives *q* often results in inaccurate calculation of charge amount, which leads to insufficient charge or residual charge during blasting, affecting the efficiency of blasting.

## The three-dimensional solid model of the blast hole charge

### Establish three-dimensional solid model of blasting area

#### Interpolate to generate 3D solid model of rock mass

Today, many scholars are devoting themselves to 3D solid modeling^25–32^. The inverse distance square interpolation method has good universality, and it is available in the case of missing strata and extremely uneven borehole distribution, and the interpolation error is relatively small, so the inverse distance square interpolation method is adopted^33^. The inverse distance square method is a kind of interpolation method related to spatial distance. When calculating the value of interpolation points, according to the principle that the closer the distance is, the greater the weight value is, the linear weighting of several adjacent points is used to fit the value of estimated points^34–36^. The calculation formula is^37^:

2 $$g = \frac{{\mathop \sum \nolimits_{i = 1}^{n} \frac{1}{{d_{i}^{p} }}g_{i} }}{{\mathop \sum \nolimits_{i = 1}^{n} \frac{1}{{d_{i}^{p} }}}},$$

where *g*—estimated value; *g*_*i*_—the ith sample; *d*_*i*_*—*distance; *p*—power of distance, its value significantly affects the result of estimated value.

The blasting area is divided into many solid units according to the given solid unit's length, width, and height, obtaining the solid element set $E_{0} = \{ e_{1} , e_{2} ,\ldots , e_{i} , \ldots e_{n} \}$ of the entire blasting area. Therein $e_{i}$ is the i-th solid unit, $i \in [1,\;n]$, *n* is the total number of solid units. Taking the blast hole lithology distribution data as a sample, use the inverse square distance method to perform lithological interpolation on each solid unit in the solid unit set $E{}_{0}$ and assign lithology to each solid unit to generate a three-dimensional rock mass entity model.

#### Cutting solid model with the polygon in the blasting area

The triangle grid is generated via the blasting range polygon, and the blasting range triangle set $T_{b} = \left\{ {t_{b1} , t_{b2} , \ldots , t_{{bi^{\prime}}} , \ldots t_{bl} } \right\}$ is obtained, where $t_{{bi^{\prime}}}$ is the *i*th triangle, $i^{\prime} \in \left[ {1,\;l} \right]$, *l* is the total number of triangles of the blasting range polygon; the blasting area triangle set $T_{b}$ is used to cut the entity unit set $E_{0}$ of the blasting area, and the 3D entity of the rock mass within the blasting area is retained, recorded as $E_{1}$.

#### Triangle cutting of solid model in stope step

Triangulate the step line of the stope^38–40^ to get the triangle set $T_{c} = \{ t_{1} , t_{2} , \ldots , t_{j} , \ldots t_{m} \}$. Therein $t_{j}$ is the j-th triangle of stope step, $j \in [1,\;m]$, $m$ is the total number of triangles for stope steps; use the stope triangle set $T_{c}$ to cut the 3D entity set of rock mass $E_{1}$ within the blasting range, and retains the 3D rock mass entity below the step triangle of the stope, namely the three-dimensional solid model of rock mass in blasting area, recorded as $E = \left\{ {e_{1} ,\;e_{2} , \ldots ,e_{{j^{\prime}}} , \ldots e_{k} } \right\}$. Therein $e_{{j^{\prime}}}$ is the $j^{\prime}$ th entity unit, $j^{\prime} \in [1,\;k],\; k$ is the total number of entity units in the blasting area after cutting.

### Calculate the influence range of blast hole

#### Establish triangle grid of blast hole points

Triangulate the blast hole position point set $P = \{ p_{1} , p_{2} , \ldots , p_{f} , \ldots p_{F} \}$ and the polygon vertices of the blasting area, then get the blast hole triangle set $T_{r} = \left\{ {t_{r1} , t_{r2} , \ldots , t_{rh} , \ldots t_{rH} } \right\}$, among them,$p_{f}$ is the position of the f-th blast hole, $f \in [1,F]$, *F* is the total number of blast hole positions in the polygon of the blasting area, $t_{rh}$ is the *h*-th divided triangle, $h \in [1,H]$, *H* is the total number of triangles obtained by triangulating the blast hole position point and the polygon vertices of the blasting area.

#### Get the blast hole influence polygon

For each blast hole, from the blast hole triangle set $T_{r}$, all the triangles connected to the blast hole position points are obtained. The blast hole influence polygon is formed by connecting the center points, the midpoints of the sides or the vertices of the triangles connected with it in sequence. The set of all blast hole influence polygons is denoted as $PL = \left\{ {pl_{1} , pl_{2} , \ldots , pl_{m} , \ldots , pl_{F} } \right\}$, where $pl_{m}$ is the influence polygon of the m-th blast hole, $m \in \left[ {1,\;F} \right]$. The acquisition method of blast hole influence polygon is as follows:

1. If the three vertices of the triangle connecting the blast hole are all blast hole points, the center point of the triangle is considered as the vertex of the blast hole influence polygon;
2. If only two of the three vertices of the triangle connecting the blast hole are blast hole points, then the midpoint of the two blast holes is taken as the vertex of the blast hole influence polygon;
3. If only one of the three vertices of the triangle connecting the blast hole is a blast hole point, then the other two non-blast hole points of the triangle are taken as the vertices of the blast hole influence polygon.

**Figure 2:**
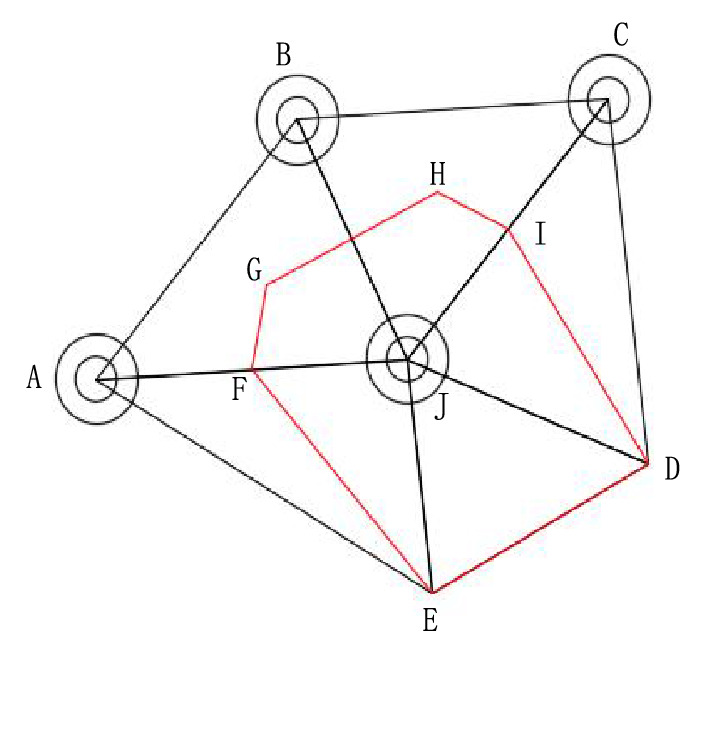
blast hole influence polygon.

Connect these vertices to form a blast hole influence polygon. As shown in Fig. 2, the influence polygon generating blast hole *J* is taken as an example to explain the method of obtaining the influence polygon of the blast hole. The points connected to the blast hole *J* are *A, B, C, D* and *E*. Among them, *A, B* and *C* are blast hole points, while *D* and *E* are non-blast hole points. The triangles connected by these vertices are ∆*ABJ,* ∆*JBC,* ∆*JCD,* ∆*JAE* and ∆*JDE*. The three vertices of ∆*ABJ* and ∆*JBC* are all blast hole points, so take the center points *G* and *H* of ∆*ABJ* and ∆*JBC* as the vertices of the blast hole influence polygon; as only two vertices of ∆*JCD* are blast hole points, the midpoint *I* of the blast hole connecting line *JC* is used as the vertex of the blast hole influence polygon. Similarly, take the midpoint *F* of the two blast hole connecting line *JA* of ∆*JAE* as the vertex of the blast hole influence polygon; ∆*JDE* has only one vertex as the blast hole point, so take the other two non-blast hole points *D* and *E* of the triangle as the vertices of the blast hole influence polygon. The blast hole influence polygon's vertices are connected to form the influence polygon of blast hole *J*, and its vertex set is $\left\{ {E,\;D,\;I,\;H,\;G,\;F,\;E} \right\}$.

### Get the blast hole influencing entity unit

The intersection of each blast hole influence polygon and the three-dimensional solid model of rock mass in the cut blasting area is used to obtain the influence solid element of each blast hole. Suppose the influence polygon $pl_{m}$ of the *m-*th blast hole is intersected with the 3D solid model *E* of the rock mass in the blasting area after cutting, and the influencing entity set $C_{m} = \{ c_{m1} , c_{m2} , \ldots ,c_{my} , \ldots ,c_{mr} \}$ of the *m*th blast hole is obtained, where $c_{my}$ is *y-*th influencing entity unit of the *m-*th blast hole, $y \in [1,\;r]$, *r* is the total number of influencing entity units of the *m*-th blast hole.

### Calculate the blast hole charge amount

According to the unit explosive consumption $q_{x}$ required by different lithologies, calculate the explosive quantity required by each influencing entity unit of the blast hole, and sum the explosive quantity required by all the influencing entity units of the blast hole to obtain the blast hole charge quantity, as shown in the following formula:

3 $$Q_{m} = \mathop \sum \limits_{y = 1}^{r} q_{x} v_{y} .$$

Among them, $Q_{m}$ is the amount of explosive required for the *m*-th blast hole, $v_{y}$ is the blasting volume of the y-th influencing entity unit, and $q_{x}$ is the unit consumption of explosives in the x-th rock formation.

## Practical application

**Figure 3:**
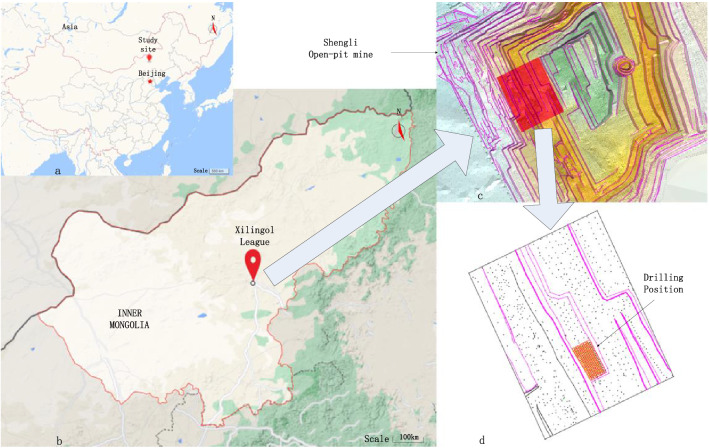
Shengli open-pit coal mine, Xilinhot, Inner Mongolia Autonomous Region, China. This figure is synthesized by Visio 2013 (https://www.microsoft.com/zh-cn/microsoft-365/previous-versions/microsoft-visio-2013), and (**a**) and (**b**) in this figure are downloaded by Google maps (https://www.google.com/maps).

**Figure 4:**
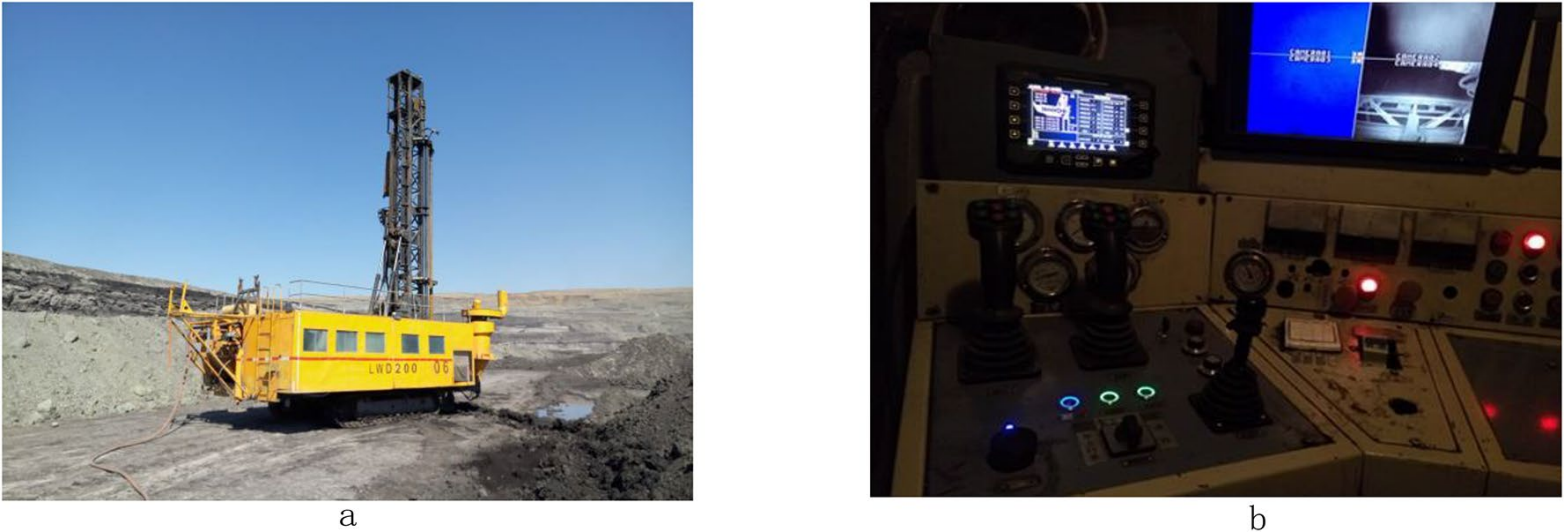
Lithology identification system of intelligent drilling rig: (**a**) LWD-200B hydraulic drilling rig; (**b**) lithology identification system.

Based on the lithology distribution data of the blasting area in Shengli open-pit coal mine, Xilinhot, Inner Mongolia Autonomous Region, China (as shown in Fig. 3), this paper establishes a three-dimensional model of the blasting area and realizes the calculation of blast hole charge amount through the calculation method of blast hole charge amount based on the three-dimensional solid model of blasting rock mass. This research is improved based on the LWD-200B hydraulic drilling rig (Fig. 4a). Considering that it has a good automation level at the beginning of the design, there remains redundant space for the transformation of the intelligent drilling rig, which significantly facilitates the intelligent transformation. The display screen of the lithology identification system communicates with the controller through the CAN bus, which can display the operation status, fault information, and lithology identification page of the drilling rig in real-time. The manual input of lithology identification number in the data acquisition test stage is also carried out through the display screen, as shown in Fig. 4b.

### Blasting data operation

#### Blasting operation scope

The blasting operation range is located in the No. 918 bench of Shengli open-pit coal mine in Xilinhot, Inner Mongolia Autonomous Region. The operation range is 125 m in length and 65 m in width, with 165 available blast holes.

#### Draw blast hole position

**Figure 5:**
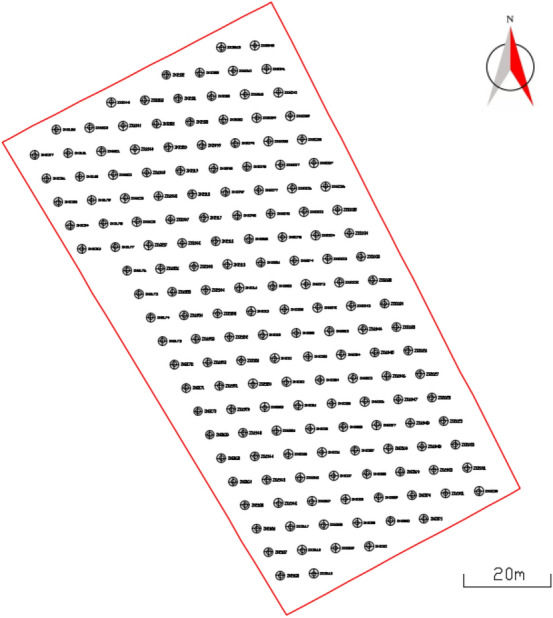
Blast hole location in operation range.

Select the range on the graph, and draw the blast holes of the selected range into the graph. The point in the graph is the location of the blast hole, and the blast holes are marked. The result is shown in Fig. 5. The blast holes are shown in the figure refer to No. 918 bench blasting blast holes in the Shengli open-pit coal mine of Xilinhot..

#### Draw the blast hole histogram

**Figure 6:**
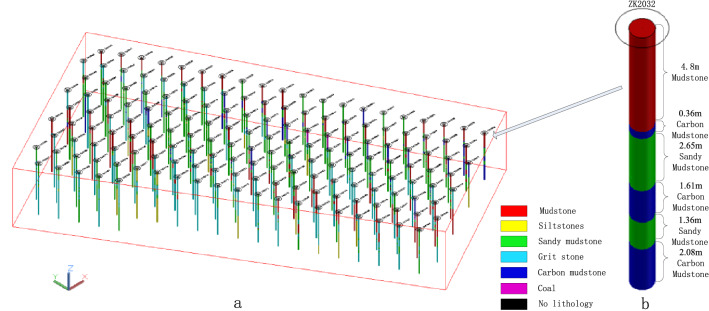
3D histogram of blasting holes: (**a**) three-dimensional histogram of all blasting holes; (**b**) ZK2032 borehole histogram.

The three-dimensional histogram is displayed in a three-dimensional form, and the structural distribution and specific thickness of the ore and rock strata inside the blast hole can be seen clearly, as shown in Fig. 6. The three-dimensional histogram of all blasting holes in the No. 918 bench of an open-pit coal mine in Xilinhot is shown in Fig. 6a; the three-dimensional histogram of a single blast hole is shown in Fig. 6b. The rocks with different lithologies are filled with three-dimensional solid of different colors. The rock thickness and lithology name are marked beside the three-dimensional rock column. The figure is the three-dimensional histogram of the ZK2032 blast hole, with a total of six layers of rocks. From the orifice to the bottom of the hole, the rock name and thickness are mudstone 4.8 m, carbon mudstone 0.36 m, sandy mudstone 2.65 m, carbon mudstone 1.61 m, sandy mudstone 1.36 m, and carbon mudstone 2.08 m.

### Establishment of 3D solid model of rock mass in blasting area

#### Interpolate 3D solid model

**Figure 7:**
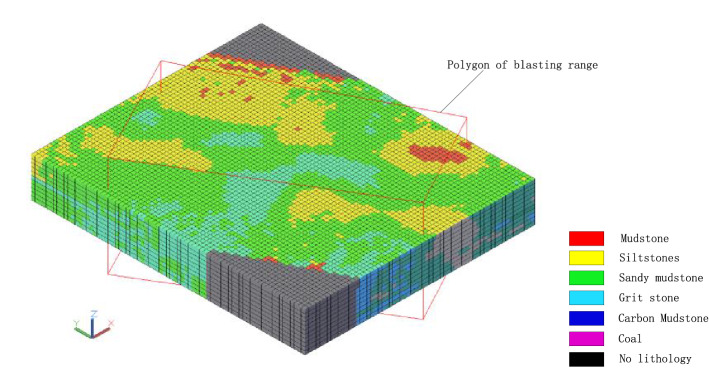
Interpolated three-dimensional solid model.

Within the blasting range, it is divided into many cube entities at a distance of 2 m (2 m in length, width, and height), and each cube entity is used as the element of the 3D solid model. The lithology data of the blast hole is taken as a sample. The inverse distance-square method is used to interpolate the lithology data of each cube element. Before interpolating, the interpolated holes should be determined according to the position of the elements, which can be divided into two steps: firstly, find all the blast holes in the range according to the searching range of 40 m; secondly, determine interpolated blast holes among blast holes obtained in the first step according to the distance from the element position from near to far and the size of the shielding angle. The inverse distance square method is used to finally complete the generation of the 3D solid model, including a total of 51,975 entities, as shown in Fig. 7. The red polygon is the blasting range polygon, and the lithology of the entities in the figure is represented by color.

#### Cutting of blasting range polygon

**Figure 8:**
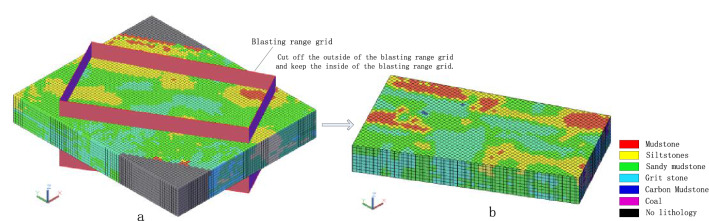
Cutting process of blasting range grid: (**a**) Blasting range grid; (**b**) 3D solid model after cutting.

Take the blasting range polygon to generate the blasting range grid (Fig. 8a), and the 3D solid model is interpolated by cutting the blasting range grid. Cut off the external entities of the scope grid, and keep the internal entities. The cutting result is shown in Fig. 8b.

#### Triangulation of grid on stope surface

**Figure 9:**
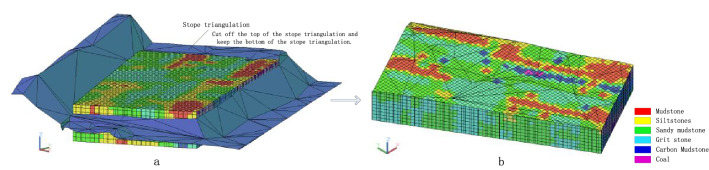
Stope cutting process: (**a**) stope triangle grid; (**b**) blasting 3D solid model after cutting.

Triangulation of the grid is operated on the bench line and measuring points of the stope to establish the triangular grid of the stope surface (Fig. 9a). Cut the blasting three-dimensional solid model with the stope triangle grid, cut off the entities above the stope triangle grid and retain the entities below (Fig. 9b). The cutting process is shown in Fig. 9. The blasting 3D solid model after cutting consists of 17,006 entities.

### Calculate the influence range of the blast hole in the blasting area

**Figure 10:**
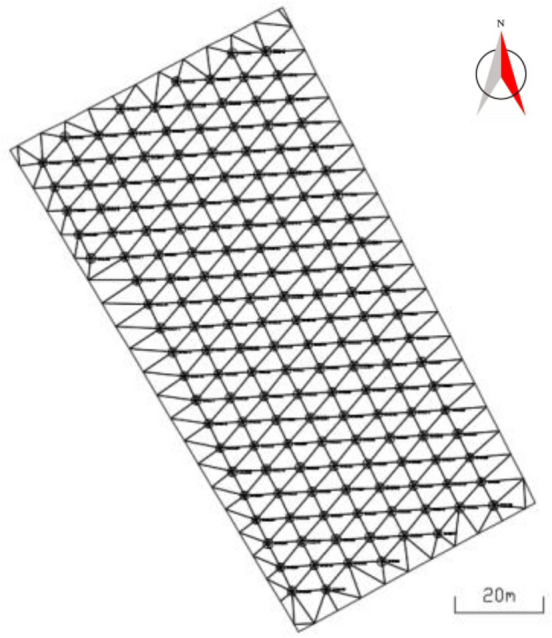
Triangle grid of blasting range.

**Figure 11:**
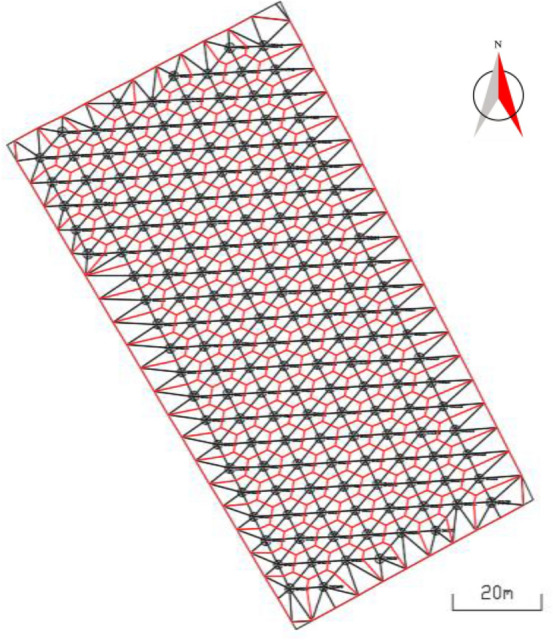
Influence range of blast hole.

There are 165 effective blast holes in this example. According to the method in 3.2, the data formed by the effective blast hole orifice position and blasting range polygon are triangulated to obtain the triangular grid of blasting range, with a total of 390 triangular surfaces, as shown in Fig. 10. The influence range of blast holes determined by each blast hole and the triangle connected with it is shown in Fig. 11.

### Calculate the influencing entity of the blast hole in the blasting area

**Figure 12:**
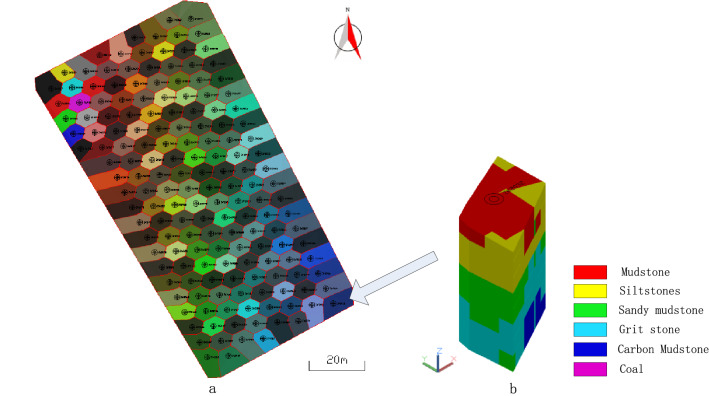
Rock column entity affected by blast hole: (**a**) the influence rock column entity of all blast holes; (**b**) the influence rock column entity of ZK2032 blast hole.

The intersection of the influence polygon of each blast hole and the 3D solid of rock mass in the blasting area is used to obtain the influence rock column solid of each blast hole, as shown in Fig. 12a,b is the influence rock column solid of ZK2032 blast hole, whose size has been enlarged in proportion.

### Calculate the hole charge amount of the blasting area

When calculating the explosive quantity required for each solid blasting, according to formula (3), the explosive quantity required for each affected solid unit of the blast hole is calculated referring to the explosive consumption required by different lithology. The sum of explosive quantity required for each solid is the explosive quantity required for each three-dimensional solid model of the blast hole.

Take the borehole ZK2032 as an example to calculate the charge amount of the borehole. The charge amount is calculated according to the volume of each entity unit and the unit explosive consumption required by different lithology. The volume and lithology of entity units of borehole ZK2032 are shown in Table 5. There are 260 solid units in total, with a total volume of 1244.521 m^3^, thus, the final charge amount of ZK2032 is 214.275 kg.

**Table 5:** Calculation charge quantity of blast hole ZK2032.

Solid unit	Volume (m^3^)	Lithology	Unit explosive consumption (kg/m^3^)	Explosive charge quantity (kg)	
1	0.069692	2	0.16		0.01115072
2	0.347538	3	0.17		0.05908146
3	0.347538	3	0.17		0.05908146
…	…	…	…		…
260	0.218819	1	0.15		0.03282285
Total	1244.521				214.275

By calculating the explosive quantity required by each affected entity unit in each hole's three-dimensional rock mass model, the explosive quantity required for each hole is obtained, as shown in Table 6. After calculation, the total charge of the blast hole is 22,849.147 kg, which is 4.59% lower than that calculated by single-hole petrology. The results show that the charge of the blast hole calculated by the three-dimensional solid model of blasting rock mass can effectively reduce the blasting cost and improve the blasting efficiency.

**Table 6:** Charge quantity of blasting 3D model calculation.

Serial number	Blast hole number	Explosive charge quantity (Kg)
1	ZK2032	214.275
2	ZK2031	205.744
3	ZK2030	198.228
…	…	…
165	ZK2027	196.445
Total		22,849.147

The above establishment application example of a three-dimensional solid model of blasting rock mass is realized by Visual C++ 2012 in detail as follows:

1. Access database operation.

ADO mode is used to connect access database to realize the operation of blast hole database.

2. Establishment and visualization of 3D solid model of blasting rock mass.

The C++ programming is used to realize the lithologic interpolation of the cube primitive, and the blasting range polygon and stope surface triangle grid are used successively to cut the blasting rock mass into a three-dimensional solid model. AutoCAD 2016 is redeveloped based on ObjectARX 2016, and the AcDbSolid class is used to visualize the 3D solid model of blasting rock mass.

With the use of the 3D solid model of blasting rock mass in calculating the charge quantity of the blast hole, the lithology of 3D solid can be adopted to calculate a more accurate charge quantity of the blast hole. In this way, the accuracy of charge quantity of blast hole and the blasting effect is improved.

## Discussion

At present, the lithology of open-pit perforation blasting is mainly obtained based on exploration boreholes. Due to the extensive distribution density and long spacing of exploration boreholes, the lithology of blasting rock mass area will not be obtained accurately. Single-hole lithology is usually used to calculate the blast-hole charge.

By comparing the blast hole charge calculated by single hole petrology with that calculated by the blast hole three-dimensional rock mass model, the results shown in Table 7 are obtained. According to the comparison, it can be seen that the charge calculated based on the three-dimensional solid model of blasting rock mass is 4.59% lower than that calculated based on single hole rock property.

**Table 7:** Comparison of blast hole charges.

Blast hole number	Single hole charge (kg)	Three dimensional model charge (kg)	Percentage of difference (%)
ZK2032	240.157	214.275	− 10.78
ZK2031	218.499	205.744	− 5.84
…	…	…	…
ZK2027	204.887	196.445	− 4.12
Total	23,948.862	22,849.147	− 4.59

## Conclusion

Based on the rock recognition data of the intelligent drilling rig, the calculation and application of the blast hole charge volume based on the three-dimensional solid model of the blasting rock mass are realized, and the conclusions are as follows:

1. Establish a blast hole database to store and manage intelligent lithology identification data;
2. Take the blast hole lithology data as a sample, adopt the inverse square distance method to interpolate the solid elements within the blasting area, and then use the blasting range polygon and stope triangle grid to cut out the three-dimensional solid model of the blasting rock mass;
3. According to the established three-dimensional solid model, through the calculation method of blast hole charge in this paper, the blast hole influence range and blast hole influence solid elements are obtained, and the blast hole charge is calculated. The charge result is compared with the charge calculated by single hole method, and the cost is reduced by 4.59%;

All the processes of calculating the blast hole charge quantity based on the 3D solid model of the blasting rock mass are realized via C++ programming. The calculation of the actual mine blasting rock mass charge amount is achieved in the application example of Xilinhot Shengli Open-pit Coal Mine in Inner Mongolia, indicating that it is helpful for the enterprise in improving blasting efficiency and reducing blasting production costs. This paper only studies the calculation of blast-hole charge. In the next step, the three-dimensional solid model of blasting rock mass can be used to optimize the design of other blasting parameters.

## Data Availability

The [xls] data used to support the findings of this study have been deposited in the [zk] repository.
